# Efficacy and cost-effectiveness of minimal guided and unguided internet-based mobile supported stress-management in employees with occupational stress: a three-armed randomised controlled trial

**DOI:** 10.1186/1471-2458-14-807

**Published:** 2014-08-07

**Authors:** David Daniel Ebert, Dirk Lehr, Filip Smit, Anna-Carlotta Zarski, Heleen Riper, Elena Heber, Pim Cuijpers, Matthias Berking

**Affiliations:** Innovation Incubator, Division Health Trainings online, Leuphana University, Rotenbleicher Weg 67, 21335 Lüneburg, Germany; Department of Psychology, Clinical Psychology and Psychotherapy, Friedrich-Alexander University Nuremberg-Erlangen, Erlangen, Germany; Centre of Mental Health and Economic Evaluation, Trimbos Institute Netherlands Institute of Mental Health and Addiction, Utrecht, the Netherlands; Department of Epidemiology and Biostatistics, EMGO Institute of Health and Care Research, VU University Medical Centre, Amsterdam, the Netherlands; Department of Clinical Psychology and EMGO Institute for Health and Care Research, VU University, Amsterdam, the Netherlands; GGZ inGeest, Regional Mental Health Service Centre, VU University Medical Centre, Amsterdam, the Netherlands

**Keywords:** Guided self-help, Unguided self-help, Occupational health, Stress management, Internet-based, Randomised controlled trial, Cost-effectiveness

## Abstract

**Background:**

Internet- and mobile based stress-management interventions (iSMI) may be an effective means to address the negative consequences of occupational stress. However, available results from randomised controlled trials are conflicting. Moreover, it is yet not clear whether guided or unguided self-help iSMI provide better value for money. Internet-based mental health interventions without guidance are often much less effective than interventions including at least some guidance from a professional. However, direct comparisons in randomised controlled trials are scarce and, to the best of our knowledge, the comparative (cost)-effectiveness of guided vs. unguided iSMI has not yet been studied. Hence, this study investigates the acceptability and (cost-) effectiveness of minimal guided and unguided iSMI in employees with heightened levels of perceived stress.

**Methods:**

A three-armed randomised controlled trial (RCT) will be conducted to compare a minimal guided and unguided iSMI with a waiting list control condition (WLC). Both active conditions are based on the same iSMI, i.e. GET.ON Stress, and differ only with regard to the guidance format. Employees with heightened levels of perceived stress (PSS ≥ 22) will be randomised to one of three conditions. Primary outcome will be comparative changes in perceived stress (PSS). Secondary outcomes include changes in self-reported depression, work-engagement, presenteeism and absenteeism. Moreover, a cost-effectiveness analysis will be conducted from a societal perspective, including both direct medical costs and costs related to productivity losses. In addition, a cost-benefit analysis will be conducted from the employer’s perspective. Incremental net-benefit regression analyses will address the question if there are any baseline factors (i.e. subgroups of employees) associated with particularly favorable cost-effectiveness when the experimental intervention is offered. Assessments take place at baseline, 7 weeks post-treatment and 6 months after randomisation.

**Discussion:**

Online-based (guided) self-help interventions could be an acceptable, effective and economically sustainable approach to offer evidence-based intervention alternatives to reduce the negative consequences associated with work-related stress. This study evaluates the (cost-) effectiveness of two versions of an iSMI, minimal guided and unguided iSMI. Thus, the present study will further enhance the evidence-base for iSMI and provide valuable information about the optimal balance between outcome and economic costs.

**Trial registration:**

German Clinical Trial Registration (DRKS): DRKS00005687.

## Background

Occupational stress has shown to be a major risk factor for a range of health outcomes, such as depression [1], coronary disease ( [2] and related mortality [3]. Moreover, stress is also associated with substantial economic costs due to absenteeism, staff turnover and reduced work performance [4].

In the last decades, a number of interventions for occupational stress have been developed for which efficacy has been demonstrated in a large number of randomised controlled trials. When comparing different types of occupational stress management interventions (SMI) it has been shown that cognitive-behavioural programmes aimed at the individual consistently produced larger effects compared to other interventions (e.g., relaxation, organisational interventions) [5–7]. However, the majority of affected individuals remains untreated [8].

Using the Internet to provide self-help interventions may help to overcome some of the limitations of traditional SMI such as limited availability, high threshold and costs. Advantages of Internet-based interventions are besides others that: (1) they are easily accessible at any time and place, (2) anonymity is assured when employees want to avoid stigmatisation or self-disclosure in group settings, (3) participants can work at their own pace and review materials as often as they want, and (4) such interventions may reach affected employees earlier than traditional mental health services, hence preventing the onset of more severe mental health problems. Finally (5), internet-based interventions are easily scalable, implying that only a small increase of therapeutic resources is required for reaching a greater proportion of the eligible population using these interventions [9–13].

Internet-based interventions have shown to be effective in community and clinical settings, including the treatment of depression [14–16], anxiety [17, 18] and sleep disorders [19]. However, only a few interventions have been developed and evaluated to address the specific needs of the working population. So far, RCTs on Internet-based SMI show conflicting results, with some studies reporting significant results with moderate effects sizes on perceived stress [12, 20] and others not finding significant results [21, 22].

Although it is often assumed that Internet-based interventions offer good value for money, evidence for their cost-effectiveness from randomised trials is still scarce and, to the best of our knowledge, completely absent when it comes to internet-based SMI in the work setting. Studies currently investigating the cost-effectiveness of mental health interventions for workers include a study on a guided self-help course for workers with depressive symptoms [23] and a study on a guided self-help regeneration training for stressed employees with work-related rumination and sleeping problems [24]. Our group recently conducted a randomised controlled trial testing the efficacy and cost-effectiveness of an internet-based SMI in employees with heightened levels of perceived stress [25]. Results are still pending. However, that latter study only evaluated an intervention including substantial professional support (up to 4 h per participant) which is thus very time-consuming and expensive. This clearly limits the possible reach of the intervention and, consequently, its potential to reduce the negative consequences of occupational stress at population level. Once developed, costs of internet-based interventions are substantially linked to professional guidance time. Thus, evaluating whether interventions with less intensive guidance up to 1 h per participant (minimal guidance, or unguided interventions) may still be effective appears promising.

Internet-based mental health interventions without guidance are often much less effective than interventions including at least some guidance from a professional [15, 16]. However, direct comparisons in randomised controlled trials are scarce. A recent systematic review identified only five of such randomised trials evaluating internet-based interventions for mental health related problems (Reichler et al. in prep). Guided interventions were significantly superior to unguided interventions (d = 0.22). Nevertheless, despite the possibly lower effectiveness of unguided interventions compared to guided interventions, unguided self-help may still produce larger effects at a population level with regard to the reduction of disease burden, given that more individuals can be reached for comparable costs. However, to the best of our knowledge, no study on the comparative efficacy and cost-effectiveness of guided and unguided SMI has been published so far.

### Aims

Thus, this study aims at strengthening the evidence-base for internet- and mobile-based SMI (iSMI) by investigating the acceptability and (cost-) effectiveness of minimal guided and unguided iSMI in employees with heightened levels of perceived stress.

The study has the following specific aims: (1) To assess the effectiveness of minimal guided and unguided versions of iSMI for reducing perceived stress when compared to a waiting list control group (WLC). (2) To assess the comparative effectiveness of minimal guided and unguided iSMI. (3) To assess the cost-effectiveness of minimal guided and unguided iSMI compared to WLC. (4) To assess the comparative cost-effectiveness of minimal guided and unguided iSMI from a societal perspective. (5) To assess the cost to benefit ratio and return-on-investment of offering the interventions from an employer’s perspective. (6) To assess and compare acceptability of minimal guided and unguided iSMI. (7) To investigate in explorative analyses if there are any baseline factors (i.e. subgroups of employees) associated with particularly favourable cost-effectiveness when the experimental intervention is offered.

We hypothesise minimal guided and unguided iSMI both to be more effective and cost-effective than WLC; minimal guided iSMI to show higher effectiveness, cost-effectiveness and acceptability than unguided iSMI. Likewise, we expect guided iSMI to represent a better business case as seen from an employer’s perspective than unguided iSMI and WLC, respectively.

## Methods

### Design

**Figure 1:**
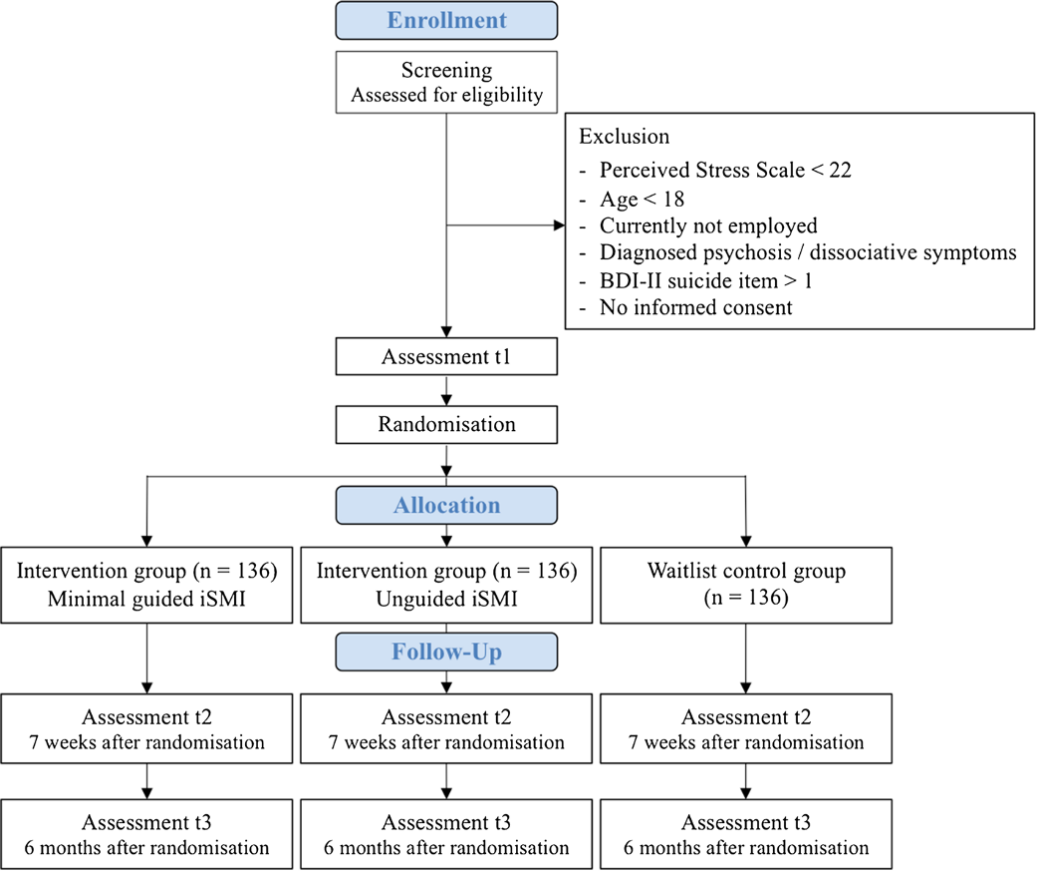
**Study flow.**

A three-armed randomised controlled trial (RCT) will be conducted to compare minimal guided and unguided iSMI (GET.ON Stress) with a waiting list control condition (WLC). All intervention arms will have full access to treatment as usual (TAU). To control for potential confounding effects, TAU will be monitored. Assessments will take place at baseline (T1), post-treatment (7 weeks, T2), and at 6-months follow-up (T3; see Figure 1 for a detailed overview of assessments). All procedures involved in the study will be consistent with the generally accepted standards of ethical practice. The study was approved by the University of Marburg ethics committee (No. 2014-5K). The trial is registered in the German clinical trials register under DRKS00005687.

### Participants & procedure

#### Inclusion and exclusion criteria

We will include a) currently employed workers b) above the age of 18 c) with scores ≥ 22 on the Perceived Stress Scale (PSS-10 [26]), d) who have internet access, e) sufficient German skills in reading and writing (self-report) and f) who are willing to give informed consent. We will exclude subjects a) reporting to have been diagnosed with psychosis or dissociative symptoms in the past, b) showing a notable suicidal risk as indicated by a score greater than 1 on BDI [27] Item 9 (“I feel I would be better off dead”).

The cut-off on the PSS was chosen to select participants with a heightened level of subjective stress as identified by one standard deviation (SD = 6.2) above the mean (PSS-10 = 15.3) in a large working population [28].

#### Recruitment

The study is integrated in the primary prevention and occupational health management programme of a large health insurance company in Germany. Participants are recruited via a) the insurer´s homepage, b) all regional health insurance offices of the cooperating insurance company (n = 918), c) advertisements in newspapers and d) an article in the members-journal (quarterly circulation 5.8 Million). Recruitment takes place between January and May 2014. Interested people can sign in for participation on http://www.geton-training.de. The research website provides information about the GET.ON stress training and details about the study. The trial is open to all individuals fulfilling the inclusion criteria and is not restricted to members of the cooperating health insurance company. People interested in participating in the study can apply online by providing the research team with their e-mail address.

#### Assessment of eligibility and randomisation

People who apply for study participation will receive an online information letter with detailed information about the study procedures and will be asked to provide an e-mail-address and a first and last name (which can be pseudonyms if desired) to participate. They will be informed that they can withdraw from the intervention and/or study at any time without any negative consequences. Applicants who continue to participate in the study will be asked to complete online screening questionnaires that assess the severity of their stress level (PSS ≥ 22), and to state whether they have a high suicidal risk (BDI Item 9 > 1) and whether they have been diagnosed with psychosis or dissociative symptoms in the past.

Participants meeting all of the inclusion and none of the exclusion criteria who have completed the baseline assessment and returned the informed consent form will enter the study and will be randomly allocated to study conditions. Randomisation will take place at an individual level. The allocation will be performed by an independent researcher not otherwise involved in the study using an automated computer-based random integer generator (randlist). During the randomisation process, allocation will be concealed from participants, researchers involved in recruitment, and eCoaches. Participants will be informed about the outcome of the randomisation and participants in the intervention group will receive immediate access to the GET.ON Stress training. However, the participants in the control group will receive the login data required to complete the training six months later than the intervention group.

#### Assessments

Self-report assessments will take place at baseline, post-intervention (7 weeks), and at the 6-months follow-up. See Figure 1 for a detailed overview. Self-report data will be collected using a secured online-based assessment system (AES, 256-bit encrypted).

### Intervention

The web-based “GET.ON Stress” intervention is based on two main components: problem solving and emotion regulation. The intervention consists of eight sessions composed of modules for psycho-education (session 1), problem solving (sessions 2–3), emotion regulation (sessions 4–6), planning for the future (session 7) and a booster session (session 8). Additionally, participants are offered 8 modules that are integrated in sessions 2 to 6 and that can be chosen based on individual need and/or preference. Additional modules are directed at time management, rumination and worrying, psychological detachment from work, sleep hygiene, rhythm and regularity of sleeping habits, nutrition and exercise, organisation of breaks during work, and social support (see Table 1 for a session overview). Each session can be completed in approximately 45 to 60 minutes. We advise participants to do at least one and maximal two sessions a week. Consequently, the training lasts about 4 to 7 weeks (plus booster session after 4 weeks). Lessons consist of texts, exercises, and testimonials and also include interactive elements such as audio and video clips. Participants are encouraged to keep a daily online stress diary. A strong focus of the intervention lies on transfer tasks (homework assignments) to integrate newly acquired strategies and techniques into daily life. The training is adaptive as the content is tailored to the specific needs of the individual participant by continuously asking participants to choose among various response options. Subsequent content is then tailored to each participant’s response. Using responsive web-design, participants can follow the programme on the internet, a tablet or mobile phone. An integrated read-aloud function allows participants to follow narrated lessons. If desired, participants will receive automatic motivational text messages and small exercises on their mobile phones. These messages will support the participant in transferring the exercises of the training into their daily lives (e.g., short relaxation exercises: “Relax your muscles in your hands and arms for 3 seconds now. Follow your breathing and each time you breathe out, relax a little more”). The participants will have the opportunity to choose between “light coach” (one text message every other day) and “intensive coach” (2–3 text messages every day) options.

**Table 1:** **Content of the GET.ON stress training**

Session	Intervention content	
1	Psycho-education	
2	Problem-solving I	Learning Phase
3	Problem-solving II	Maintenance Phase
4	Emotion regulation I	Muscle- and breathing relaxation
5	Emotion regulation II	Acceptance and tolerance of emotions
6	Emotion regulation III	Effective self-support in difficult situations
7	Plan for the future	Reflection on goal attainment and learning experiences. Implementation intentions until booster session
8	Booster session	Reflection on goal attainment and learning experiences. Implementation intentions for the coming months

### Support

#### Unguided iSMI

Participants of the “unguided” treatment-arm have contact with the study-administration team during the study period and are not supported by an eCoach. They are provided, however, with an e-mail address which can be contacted in case of any technical problems.

#### Minimal guided iSMI

Participants of the “minimal guided” treatment-arm are supported by an accompanying eCoach. The guidance manual is mainly based on the supportive-accountability model of providing guidance in internet interventions [29]. In this model, it is argued that adherence to an Internet-intervention (and thereby effectiveness) can be enhanced via human support through accountability to a coach who is seen as legitimate, trustworthy, benevolent, and having expertise. In the current study, the purpose of the guidance will thus be to support participants to adhere to the treatment modules but will be kept to a minimum to minimise costs. Every participant will be assigned to one eCoach throughout the study. The eCoaches are trained psychologists and will follow guidelines about the feedback process that are defined according to the standardised manual for the intervention. Coach guidance consists of two elements: (a) adherence monitoring and (b) feedback on demand.

*Adherence monitoring* includes offering participants to support them to adhere to the intervention by regularly checking whether participants have completed intervention sessions on time, and if not, to remind them to do so. The eCoaches will send reminders in case that participants did not complete at least one session within 7 days. Both, personal and automatic reminders have shown to improve adherence to self-guided health promotion and behaviour change interventions [30, 31], but in the model of supportive accountability, it is assumed that personal reminders from a coach are perceived as benevolent and are more effective than automatic reminders. According to the model of supportive accountability, it is made clear to the participant that the aim of adherence monitoring is to provide feedback and that feedback in turn provides opportunities for self-reflection, thus aiming to help to achieve personal goals rather than exposing or punishing the participant.

*Feedback on Demand* includes offering participants the opportunity to contact the coach via the internal messaging system of the platform and receive individual support/feedback whenever such a need may arise. Within 48 hours, the participants will receive personalised written feedback. In contrast to other guidance concepts, coach guidance will only take place on initiative of participants. Feedback is not assumed to directly have an influence on the effectiveness of the intervention. Instead it aims at creating perceived legitimacy of the coach and a sense that the coach has the participant’s best interest at heart [32]. It is assumed that people respond more positively to adherence demands from a coach who is perceived as legitimate [29, 33]. Hence, perceived legitimacy of the coach is assumed to further increase adherence to the intervention and to be a necessary precondition that adherence monitoring will have positive effects.

### Primary and secondary outcomes

Primary outcome will be perceived stress at post-treatment. In secondary analyses, we will explore the effects of the intervention on depression, anxiety, emotional exhaustion, quality of life, absenteeism/presenteeism and numbers of participants displaying a reliable positive change. Economic analyses will be conducted assessing cost-effectiveness and cost-utility from a societal perspective including the costs of all types of health services and the costs that stem from productivity losses. Additionally, cost-benefit analyses from an employer’s perspective will be conducted. In explorative analyses we will also investigate whether there are any baseline factors (i.e. subgroups of employees) associated with particularly favorable cost-effectiveness when the experimental intervention is offered.

### Measures

#### Perceived stress

The German version of the ten-item-Perceived Stress Scale (PSS-10) [26, 34] will be used as a primary outcome measure. The PSS is “the most widely used instrument for measuring perceived stress” and assesses the degree to which people perceive their lives as stressful, particularly regarding how “unpredictable, uncontrollable and overloading respondents find their lives” [35]. Cronbach’s alphas range for this scale from .78 to .91 [35]. The scale is based on Lazarus’ transactional model of stress and, therefore, fits well with the theoretical basis of the intervention. Participants in this study will be asked to answer questions relating to the past week as opposed to the past month to avoid confounding with the training period. Similar procedures have been adopted in previous studies [34].

#### Depression

Depressive symptoms will be measured with the short version of the German version of the Center for Epidemiological Studies’ Depression Scale (CES-D) [36–38]. This frequently used self-report instrument consists of 15 items that are answered on a four-point Likert scale referring to the previous week. Total scores range from 0 to 60. The internal consistency of this measure has been found to be excellent (α = .95) [39].

#### Emotional exhaustion

The German version of the Maslach Burnout Inventory (MBI-GS-D) [40, 41] will be used to measure emotional exhaustion, the basic stress dimension of burnout. This commonly used self-report instrument consists of five items and uses a six-point Likert-type scale anchored by 1 = “never” and 6 = “very often”. The internal consistency of this subscale was α = .85 in a German sample [41].

#### Work engagement

The Utrecht Work Engagement Scale (UWES) [42] assesses work engagement defined as a “positive, fulfilling, work-related state of mind that is characterised by vigor, dedication, and absorption” [43]. This scale has nine items, and the internal consistency of the total score is α = .91 [42]. The reference period involves the last two weeks.

#### Quality of life

We will use the Short Form 12 (SF-12) [44] and the (EuroQol EQ-5D) [45] to assess quality of life. The SF-12 [44] covers eight health domains (physical functioning, role limitations, pain, general health perception, vitality, mental health, emotional role and social functioning) and allows for the calculation of two sum scores for physical and mental health. We will also use the EQ-5D which is a widely applied, valid and reliable measurement of quality of life and consists of five items related to mobility, self-care, common activities, pain/discomfort and anxiety/depression. Furthermore, this measurement contains a visual analogue scale concerning health state valuation. The EQ-5D is only assessed for use in the health economic evaluation and is no secondary outcome itself.

#### Cost measure

Information on the participants’ use of health services will be obtained with the German Version of the Trimbos and institute of Medical Technology Assessment Cost Questionnaire for Psychiatry (TiC–P-G; [46]). With this questionnaire participants register the number of “work loss” days (absenteeism from work) and the number of “work cut-back” days (reduced efficiency at work while feeling ill), general practice visits, sessions with psychiatrists, hospital days, etc. will also be measured with help of the TiC–P-G. The questionnaire has shown to have a good retest-reliability and to achieve comparable results between patient-reported data and data derived from medical registrations [46].

#### Course evaluation

In absence of a standardised measure for evaluating course satisfaction in internet-based treatments, user satisfaction will be measured with a self-designed questionnaire based on the Client Satisfaction Questionnaire (CSQ-8 [47], German Version [48]). This self-report scale consists of 8 items measuring the global client satisfaction with the internet-based training. Previous research indicated a high internal consistency [48].

#### Response

To determine the numbers of participants achieving a reliable positive outcome we will code participants as responders or non-responders according to the widely used reliable change Index (RCI [49]). Participants will be considered responders, when they display a RCI score of above 1.96. To determine potential negative effects of the intervention on course of symptom [50] we will also report number of participants with reliable symptom deteriorations according to the RCI [51].

#### Other measures

Other measurements include demographic variables (e.g., age, gender, occupation etc.), the Effort Reward Imbalance Questionnaire – Short Form (ERI-SF) [52], the emotion regulation skills questionnaire (ERSQ) [53, 54]. Big Five Inventory (BFI-10) [55], Volitional Components Questionnaire – Short Form [56], General Self-Efficacy Scale [57], Self-Regulation Scale [58], Self-Control Scale (SCS-K-D) [59, 60], Psychotherapy Motivation Questionnaire – Short Form [61, 62], Health Action Process Approach Questionnaire (HAPA-Questionnaire) (according to the guidelines by [63]), Internet Affinity Questionnaire [64] and the Connor-Davidson Resilience Scale (CD-RISC) [65]. To assess presenteeism, we used the Work Limitations Questionnaire (WLQ-8) [66], the Single-Item Presenteeism Question [67] and a single item question on work ability [68]. Approximately 40 minutes will be required to complete all questionnaires at baseline, and approx. 20 minutes at follow-ups. For an overview of all outcome measures, see Table 2.

**Table 2:** **Measures**

	T0	T1	T2	T3
Perceived stress scale	✓	✓	✓	✓
Center for epidemiological studies depression scale	-	✓	✓	✓
Maslach burnout inventory – emotional exhaustion	-	✓	✓	✓
Emotion regulation skills questionnaire – general distress	-	✓	-	-
Utrecht work engagement scale	-	✓	✓	✓
Trimbos/iMTA questionnaire for costs associated with psychiatric illness	-	✓	-	✓
EuroQol, SF-12 quality of life	-	✓	-	✓
Other measurements				
Demographic variables questionnaire	✓	-	-	-
Effort reward imbalance questionnaire – short form	-	✓	✓	✓
Client satisfactory questionnaire	-	-	✓	-
Potential dropout reasons	-	-	✓	-
Big five inventory – short form	-	✓	-	-
Volitional components questionnaire – short form	-	✓	-	-
General self-efficacy scale	-	✓	-	-
Self-regulation scale	-	✓	-	-
Self-control scale	-	✓	-	-
Psychotherapy motivation questionnaire – short form	-	✓	-	-
HAPA-questionnaire	-	✓	-	-
Internet affinity questionnaire	-	✓	-	-
Work limitations questionnaire	-	✓	✓	✓
The single-item presenteeism question	-	✓	✓	✓
A single item question on work ability	-	✓	✓	✓
Connor-Davidson resilience scale	-	✓	✓	✓

Note. T0 = Screening, T1 = Baseline, T2 = 7 weeks, T3 = 6 months.

### Sample size calculation

We aim to include 408 participants. The study was designed based on the expected superiority of the active groups (minimal guided and unguided internet-based self-help) compared to the waiting-list control group on the primary outcome variable (i.e. perceived stress) at post-test. Based on pilot-evaluation data we expect a mean effect for the minimal guided treatment arm compared to the waiting list control group of d = 0.70. However, unguided internet-based interventions consistently produce lower effect sizes as guided interventions [15] and a meta-analysis on traditional face-to-face interventions for work-related stress [6] yielded an overall combined effect size of only d = 0.34. As we also want to examine the difference in the effectiveness of minimal guided and the unguided iSMI we aim to be able to detect difference between groups with an effect size of d = 0.30. Thus we need to include 408 participants. This sample will allow us to detect a between-group effect size (ES) of d = 0.30 with a power (1-ß) of 80 % and an alpha of .05 (calculated using PASS 12) in a one-tailed test (for uni-directional hypotheses H1: WLC < unguided < minimal guided iSMI).

### Statistical analyses

#### Clinical effectiveness

The trial will be conducted in compliance with the study protocol, the Declaration of Helsinki and GCP. Aiming at an intention-to-treat design we will include all participants who will be randomly assigned to conditions. Additionally, per protocol analyses (PPA) will be conducted, including only participants’ satisfying protocol treatment. Analyses of variance will be conducted to explore the effects of the treatments on all primary and secondary outcomes. Missing data will be handled using multiple imputations (MI). MI is especially robust with respect to missing data [69]. For all analyses, Cohen’s d [70] will be calculated by standardising the differences between baseline and follow-up scores by the pooled standard deviation of the baseline scores. We will also calculate the number needed-to-be-treated (NNT) with GET.ON Stress minimal guided and unguided to achieve one response compared to the control group. For all statistical analyses, significance level will be set at p < .05, one-sided. The effectiveness evaluation will be conducted in accordance with the CONSORT statement [71].

#### Cost-effectiveness and cost-utility analyses

In the cost-effectiveness analyses, treatment response (reliable positive change) will be the primary outcome, whereas quality adjusted life years (QALYs) will be the outcome in the cost-utility analyses. A cost-effectiveness acceptability curve [72] will be calculated, when better outcomes are associated with higher costs. Such an acceptability curve represents the probability that the intervention is cost-effective relative to the control group, given varying thresholds for the willingness to pay (WTP) for gaining one additional treatment responder or gaining one additional QALY. Nonparametric bootstrapping will be applied to estimate confidence intervals for mean differences in costs and effects between groups.

#### Cost-benefit analysis from the employer’s perspective

As indicated, a cost-benefit analysis will be conducted from the employer’s perspective. Costs, C, will be equated with intervention costs; benefits, B, with greater productivity owing to lesser absenteeism and lesser presenteeism. The ratio C/B is the cost-benefit ratio, and its inverse, B/C equals the return-on-investment, while the net-benefit is defined as B-C, which is the payout of offering the intervention. All health-economic evaluations will be conducted in accordance with the CHEERS statement for reporting health economic studies [73]. Sensitivity analyses directed at uncertainty in the main cost-drivers will be conducted to ascertain the robustness of the outcomes.

#### Incremental net-benefit regression analysis

Effect modification can be studied in the context of a health-economic evaluation to address the question if there are any baseline factors (i.e. subgroups of employees) associated with particularly favourable cost-effectiveness when the experimental intervention is offered. The data analytic approach taken here consists of incremental net-benefit regression analysis (INBRA). First, incremental net-benefits, Δ(NB), are calculated as *Δ*(NB) = WTP × *Δ*(QALY) - *Δ*(Costs), where WTP × Δ(QALY) is the willingness to pay for gaining one QALY and Δ(Costs) are the extra costs owing to offering the intervention. Δ(NB)s are calculated for each participant in the dataset and can then be analysed in a regression framework. In its simplest form the incremental net-benefits are regressed on the treatment indicator variable to evaluate the cost-effectiveness of the intervention relative to the control condition. When Δ(NB) exceeds €0, then the intervention is deemed to be cost-effective. The regression models can be expanded to include interaction terms of the treatment indicator variable and putative effect modifiers [74, 75].

## Discussion

Occupational stress has shown to be a major risk factor for a range of health outcomes, and is moreover associated with substantial economic costs. Online-based (guided) self-help interventions could be an attractive, efficient and cost-effective approach to offer evidence-based intervention alternatives to reduce the negative consequences associated with work-related stress. However, results on iSMI are still conflicting. Moreover, it is unclear whether guided or unguided self-help approaches would provide better value for money when implemented at a large scale. The marginal costs of treating a person with a pure unguided Internet-intervention will get smaller and smaller when more people would make use of the e-health intervention alone. However, the costs of guidance by a professional coach are fixed and remain the same for every other person being treated. Thus, research on the dose–response relationship (e.g. guidance yes/no) is one of the most important research questions from an economic viewpoint. However, to date, the response–dose relationship has not been examined for iSMI nor for other internet-based occupational health management interventions.

A previous study on GET.ON Stress evaluated an intervention version with weekly guidance from an accompanying eCoach [25]. This study now evaluates the acceptability, comparative efficacy and cost-effectiveness of two more economic versions of support in iSMI, namely minimal guidance and no guidance. Thus, the present study will provide valuable information about optimal pay-off between treatment outcome and economical costs necessary before wide dissemination of such interventions.

This study will also have some limitations. First, this study does not include any objective measurement of stress (e.g. cortisol levels). Due to feasibility limitations, only self-report measurements will be examined. Although self-reports always carry the risk of introducing subjective biases, it has been suggested that replacing self-reports with stress-related physiological measurements is not promising [76]. Second, we only include participants experiencing heightened levels of perceived stress, as indicated by a cutoff score of ≥ 22 on the Perceived Stress Scale. Thus, our results will only be generalisable to employees fulfilling this criterion. In occupational health routine practice SMI are often offered to the whole working population (i.e. universal prevention approach) instead of selecting participants based on baseline stress level. Therefore, future studies should evaluate this iSMI using a universal prevention approach to allow for a reliable estimation of efficacy and cost-effectiveness of the intervention for this target group. Finally, the study is powered to detect differences between groups in the reduction of perceived stress. Hence, the sample size will be too small to draw reliable conclusion for potential moderating effects between guided and unguided groups with regard to the clinical effectiveness of the intervention. Which participants are thus likely not to profit from unguided but from minimal guided iSMI will remain unclear. However, health-economic evaluations do not test hypotheses. Instead, a probabilistic decision-making approach is undertaken. Thus, this study will address the question “who benefits most?” in explorative analyses by relying on an incremental net-benefit regression analysis.

There will also be several strengths of this study, including the randomised controlled design with the direct comparison of two active conditions, an appropriate statistical analysis plan and handling of missing data with state of the art methods. Moreover, to the best of our knowledge, direct comparisons of the costs and benefits of different levels of support in internet-based occupational health management interventions have not been published so far.

Overall, to overcome the gap between the need for effective strategies for managing negative consequences of occupational stress and evidence-based treatment availability/utilisation, (cost-) effective low-threshold interventions are needed that are accessible for as many people as possible. Internet-based occupational health interventions might be a promising strategy to overcome some of the limitations of face-to-face occupational health interventions. This study will enhance the evidence-base for iSMI and provide information about the differential acceptability, efficacy and cost-effectiveness of two economic support forms in iSMI.
